# Palliative care in its own discourse: a focused ethnography of professional messaging in palliative care

**DOI:** 10.1186/s12904-020-00582-5

**Published:** 2020-06-22

**Authors:** Carla Reigada, Maria Arantzamendi, Carlos Centeno

**Affiliations:** 1 ATLANTES Research Group; Institute for Culture and Society, University of Navarra, Campus Universitario, 31009 Pamplona, Spain; 2 Health Research Intitute of Navarra (IdiSNA), Pamplona, Spain; 3 Palliative Medicine Department, Clinica Universidad de Navarra, Pamplona, Spain

**Keywords:** Palliative care, Message, Ethnography, Daily practice, Health professionals, Patient-professional interaction

## Abstract

**Background:**

Despite 50 years of modern palliative care (PC), a misunderstanding of its purpose persists. The original message that PC is focused on total care, helping to live until the person dies, is being replaced and linked to feelings of fear, anxiety and death, instead of compassion, support or appropriate care. Society is still afraid to speak its name, and specialized units are identified as “places of death” as opposed to “places of life” meant to treat suffering. This issue is prohibitive to the implementation and development of PC policies worldwide. It is imperative to identify what message PC professionals are relaying to patients and other health care specialists and how that message may condition understandings of the right to access PC.

**Methods:**

A qualitative study, employing focused ethnography and participant observation (PO) of the daily interaction of PC professionals with patients and family members in three different PC services. Two researchers independently conducted a thematic analysis, followed by member checking with participants.

**Results:**

A total of 242 h of participant observation revealed the following messages sent by PC professionals in their daily interaction with patients and families: i) We are focused on your wellbeing; ii) You matter: we want to get to know you; iii) Your family is important to us.

**Conclusion:**

The complexity of PC discourses contributes to the difficulty of identifying a clear universal message between PC professionals, patients and families. The PC professionals observed transmit a simple message focused on their actions rather than their identity, which may perpetuate some social/cultural misunderstandings of PC. It seems there is a common culture, based on the same values and attitudes, within the messages that PC professionals transmit to patients and their families. PC teams are characterised by their availability.

## Background

A number of experts recommend the integration of palliative care (PC) across Europe in order to enhance its visibility and dissemination, as the “low profile” of PC may contribute to misconceptions about or unawareness of the field [1–3]. The term “Palliative Care” can evoke compassion, dignity or quality of life [4]. Already in the ‘60s, Palliative Care was presented by Dame Cicely Saunders, as the new field of care for people with progressive diseases. She launched PC as a total care approach focused on multidimensional aspects. For each severe patient’s particular situation, physical, psychological, social, emotional, and spiritual quality care should be provided [5]. However, for many, the PC message is more associated with terminal illness and death [4].

A misunderstanding of PC persists among the general public, healthcare professionals and policy makers, revealing the problem of knowledge transfer that adequately reflects reality [6, 7]. A study that analysed 600 articles from Spanish newspapers (2009–14) concluded that the information regarding PC does not accurately reflect clinical practice and its nature. Discussions of PC in the news media are usually provided by politicians in a particular context - “The Minister (from Spanish Socialist Workers’ Party,) mentioned the ‘ethical obligation’ of improving the quality of and access to palliative care” - and are characterised by strong ideological and moral content focusing on the social debate [6]. This discourse has been identified by studies as a barrier to the implementation of PC.

Furthermore, a phenomenological study by Collins et al. explored patients’ views of PC communication [8]. Thirty eligible patients eligible were asked to discuss their understanding of PC and their perception of its explanation and language use. Participants said that those who try to explain PC tend to avoid the terms “death” or “dying” and “palliative care”. Also, for these patients, the word PC is a euphemism for “my death”. Patients often feel that this concept is not articulated by the team. Professionals intend to “kindly” protect patients, but others said that even if they heard the word “palliative care”, they would not know its meaning [8, 9]. The clarity of the message conveyed by PC professionals may contribute to misunderstandings and lack of awareness. It is important that the PC message can be transmitted in a positive way by talking more about what it is and less what it is not.

In a modern society, where patients and carers are increasingly involved in clinical decision making, PC professionals have the responsibility to pass on a positive yet truthful message [10]. Therefore, it is imperative to identify what message is being transferred to patients and caregivers that may condition understandings of and access to PC. We wanted to study behaviours, attitudes and beliefs within PC teams using an ethnographic approach. Although complex and challenging, this method (originated in anthropology) is increasingly recognized as a valuable methodology in healthcare research, making it a reliable way to produce knowledge [11]. The fundamental question of focused ethnography is “What is going on here?”: a powerful approach that allows to understand the way of being of a particular community of practice and their customs [12]. By studying the communicative culture of a team, we are opening up knowledge about the organizational culture of institutions and services.

We hypothesize that a deep understanding how these professionals transmit PC values to patients and caregivers may aid the development of targeted communication strategies to disseminate a more accurate understanding of PC in the future. This study aims to understand what message is conveyed by PC professionals, explicitly or implicitly, in their daily clinical practice.

## Methods

### Study design

A focused ethnography was conducted to allow for a deep understanding of a particular problem in the palliative care context, exploring its cultural aspects, values and beliefs. Focused ethnography is a novel methodology, an adaptation from the traditional ethnography to produce research evidence of a specific practical problems in a timely manner, within a specific group [13, 14]. This study was focused on the culture of palliative care providers, not intending to characterize different professions within a team, but their overall behavior.

### Context and participants

According to the criteria of the Spanish Association for Palliative Care (SECPAL), Spain has 196 PC services (e.g. hospital care services, homecare, outpatient, mixed). This study was conducted in three provinces of the north of Spain, where we identified a total of 24 complete teams providing PC services (physicians, nurses, psychologists and social workers). Criteria for inclusion included: being recognised as PC service provider in the SECPAL directory, having at least 80% of their professionals trained in PC, and consenting to observation of their daily routine for at least 20 h per week.

Once the inclusion criteria were considered, three services were selected to cover different types of regimens. The participating services were: one service within the public system (set of free health services for the entire population); one private service operating in tandem with the public system (private health services that work with the public system and provide public free care); and one service in a private hospital (private health services for private insured patients). All PC services provided hospital care (consultation within an acute hospital), home care (home care assistance), inpatient unit care (PC beds in acute hospitals) and external clinic care (outpatient consultation).

### Access

PC team directors were contacted by researchers by email (CR, CCC), followed by meetings with the entire PC team to explain the purpose of the study and outline the observation period needed to achieve these objectives. Written consent was obtained from each team’s directors and professionals on an iterative basis [15, 16]. As the study entailed observing patient-health professional interaction, the PC team always requested permission from the patient/family prior to the observation of consultations. The researcher was introduced to patients and families as a member of the team.

### Data collection

Data were collected from February to September 2018 through participant observation and some informal conversations. Researcher’ clinical background (PC social worker) and the appointment of a “contact person” to each team facilitated rapport and smooth integration of the teams.

Each observation period commenced at the beginning of the working day of a particular PC service, which in all cases commenced with a team meeting. Each team was observed by the same researcher (researcher CR). She spent the morning (from 9 am to 2 pm) with each team because it was the period with the most clinical activity, including inpatient and outpatient consultations. At times, the afternoon was used to make notes and to consult the internal documents of the PC service team (as admission sheets, clinical protocols). Each observer rotated professionals to observe a wide variety of interactions.

Field notes were recorded using mobile phones and, later in the day, registered in more in detail on a Microsoft Word document to avoid memory bias (CR). Informal conversations conducted with professionals were also recorded in field notes (CR). Informal conversations emerged to clarify aspects observed by the researcher during specific situation. Questions like “Do you think you should introduce yourself as a Palliative Care team?” or “What do you think you convey when you give to the patient your direct contact?” were made so that the professional’s intention was clearly reported. A reflexive diary was used to reflect on the observation process and track analytic concepts. Finally, discussions were held with the research team and researchers followed up (CR, MA, CCC), where appropriate, through informal conversations with observed health professionals.

### Analysis

A thematic analysis of field notes and internal documents (e.g. team contact card) was conducted with the following research question in mind: “What is the message about PC that these professionals transmit to patients and caregivers in their daily practice?”. This helped to focus the analysis on the team-patient-family interaction more than in specific clinical interventions of care (e.g. medical or psychosocial care).

The analysis started with conversations between researchers held concurrently with data collection but was only formally initiated after data collection. There were no predefined codes. The analysis was developed inductively [17]. Field notes were coded independently by two researchers (CR and MA) who discussed the coding that each one had assigned, paying attention to differences. In the case of variances, they held further discussions to review their field notes and discuss the comments, behaviours and attitudes of each interaction described. Later the researchers started to sort codes into categories and possible themes.

The findings were presented to the participant PC services in order to obtain their feedback and enhance trust. A feedback session was conducted with each PC service at an arranged time so that the greatest number of team professionals could attend. These sessions were audio-recorded and reviewed prior to the final presentation of findings.

### Ethics approval

This study was approved (No. 2018.009) by the Ethics Committee of the University of Navarra (UNAV). This document facilitated the approval of the three institutions involved in the study. All participants provided their consent for the study.

## Results

### Characteristics of field work

A total of 242 h of participant observation (PO) with 20 PC professionals (physicians = 10, nurses = 6, psychologists = 3, social workers = 1) was carried out between February and September 2018, focused on 3 PC teams in three different regions of Spain. The majority of patients were hospitalized with an oncological disease (in the palliative care unit or elsewhere within the hospital). “Kiri”, “Oak”, or “Almond” are code names for each site. All data has been de-identified (Table 1).

**Table 1:** Key messages that Palliative Care professional convey on their clinical interaction with patients and families

Message 1: We are a team: focused in your wellbeing	Message 2: You matter: we want to meet you as a person	Message 3: Family matters: they are also importante to us
a) **We are a multidisciplinary team**	a) **We want to know about you as a person**	**a) We are here to relieve the suffering of the family**
- Multidisciplinary team to attend your holistic needs	- Life moments/experiences	- We help to resolve problems
- We show more what we do (actions), than what we are (identity)	- We recognize you as the person you are	- We want to support family too
- We tend not to use the term “palliative care” directly. We use it carefully		
b) **We are experts in symptom control**	b) **We want to know about your experience with the disease**	**b) We want to support the caregivers**
- We use pain assessment tools to measure, (re) evaluate, and prevent pain and other symptoms	- Disease trajectory	- To take care of the patient
	- Symptoms	- To take care of themselves
- It is easier to start the conversation based con symptoms control	- Clinical decision making	

PC professionals convey three central messages during their daily clinical interaction with patients and families: i) We are a team focused on your wellbeing; ii) You matter: we want to get to know you; iii) Your family is important to us.

### Message 1 – We are a team: focused in your wellbeing

The first message conveys PC professionals’ aim to improve patients’ wellbeing and includes two sub-themes: “We are a team interested in your wellbeing” and “We are experts in symptom control”.

#### We are a multidisciplinary team

All professionals were observed introducing themselves to the patient and family as a team working together to meet a patient’s needs. The work dynamic was congruent with the idea of being a team (e.g. daily team meeting will all members). The idea of the team was seen in subsequent, direct speeches, usually using the first-person plural (e.g. we are; we do), as shown in the following field note.

*(The PC team has a young hospitalized woman)*

*In addition to all anxiety that the patient presents, it does not seem that there is an excellent family relationship. The team decides that its psychologist is going to visit her today. "Hello, how's your day today? Dr A, the doctor of our team told me about you. I am the team psychologist, and I would love to chat with you for a while. Is that alright?"* (Kiri, inpatient service)

The focus of PC professionals on patients’ wellbeing was ever-present, even in the wording that they chose to use in patient interactions. The expression “Palliative care team” was used selectively.

*The nurse talks with the daughter in the office and comments that****she is part of a palliative care team****. The nurse speaks with caution and strengthens the positive aspects of her job by highlighting that they take care of people who can live for many years with their diseases. The daughter replies, "I know what palliative care is; it is true that you are associated with the subject of death but I know that you do much more."* (Oak, inpatient service)

On the other hand, though it was common to observe professionals answering phone calls with “Palliative Care team”, the team explained it was not always considered adequate to use this term during patient-team conversations. Their overall aim is the patient’s wellbeing and listening without interruption to whatever a patient may want to tell them. Situations were commonly observed where professionals emphasized their availability to meet patients’ needs over revealing their identity as PC professionals.

Professionals also highlighted their availability as a team handing in business cards with their contact details where it could be read “Palliative Medicine department”. They used these cards when explaining the patient/family that they were available to address any problem, issue or question, a pattern of behaviour congruent with the desire to promote a patient’s wellbeing. The focus on meeting patients’ needs and providing comfort was obvious when health professionals shared their awareness of the lack of use of the term “palliative care”. Their greater concern with getting to know patient needs and troubles is shown in field notes.

*“To me, the name does not worry me much. What matters to me is knowing about the patient's needs. What bothers the patient. Here, I do not insist on saying the word [PC]...My concern is to find what troubles the patient; I have a more open approach in terms of discourse to know their fears, what concerns them [the comment of the doctor].”* (Almond, inpatient service)

#### We are experts in symptom control

Together with the idea of a team, they convey that they can help control symptoms during acute situations and prevent possible future symptoms. It was common to observe interactions in which they used expressions as “We are here, so you do not have pain” (Oak), transmitting a very assertive message. The team tended to followed up the patient to ensure that there was no symptom distress. They also communicated a proactive attitude and behaviour toward symptom control, expressing directly their intention to take care of the patient, even in cases with no acute symptomology. It was observed that professionals frequently assessed symptoms with different scales, re-evaluated and checked with patients to prevent symptoms.

*“Good morning, do you remember me? I'm the nurse of (doctor's name) team. How do you feel right now? (...) We would like to register your pain (...) do you mind if we do the same exercise as last time? ( … ) So, from 0 to 10, how is your pain?”* (Kiri, outpatient consult)

The discussion of the findings on this first level - introducing message - with professionals showed that they agreed. They highlighted it was crucial for them to reach the patient, to improve the clinical situation and not to cause any more suffering. Within this, the team unanimously justified their avoidance of the term “palliative care” in case it would not be well received by the patient. They shared the idea that the most important thing was to demonstrate their ability to solve problems. This was an unwritten shared belief, as illustrated below, and was in line with observed behaviours.

*“It has to be something progressive. Our presentation is the gateway, and it can be done through symptoms or not. We have experience of being rejected when we want to get deeper into the speech... We have to go in slowly.”* (Oak, outpatient consult)

### Message 2 – you matter: we want to meet you

The second message highlights the personhood of the patient and is comprised of two sub-themes: “We want to know about you as a person” and “We want to know about your experience with the disease”.

#### We want to know about you as a person

Professionals facilitated conversations with their patients to listen to their experiences and life stories. This helped them better understand their patients with the aim of helping them. They sometimes said explicitly:

*“Is there anything I should know about you? How can we help?” (PC professional)*

“*To live!” (patient responds, then laughs)* (Kiri, outpatient consult)

Health professionals were observed dedicating time to their patients, with no sign of haste, even if later they were often seen walking quickly along the corridors due to a tightened timeframe. An essential part of patient consultations involved creating a space for the patient to share emotions and experiences. Professionals’ behaviours and conversations with patients conveyed availability, unconditional acceptance, welcoming and active listening towards the person, despite his or her limitations due to illness. The field notes illustrate how the professional stoops to his knees to stay at the same level as the patient and recognizes him or her as a person, linking back to something shared in a previous consultation.

*The team is visiting a patient at home. They know that she has had difficulty sleeping. The doctor gets on his knees by the bed and asks the patient:*

*“How are your flowers?” (the patient was a florist by profession, and she has a garden at home that she is very proud of)*

*“Ahh!!!!” (sighs, smiles) “Very well!” (responds the patient)*

*“No, no. I'm not talking about these flowers [points to the garden]. I'm talking about the most beautiful flowers you have, your daughters.”*

*They start to talk about her daughters, her family, her needs and the support she may need.* (Oak, home care service)

It was common to observe the professional approaching the patient through something related to their personhood – not their disease – and addressing a patient’s profile and experiences, promoting a symmetrical person-to-person relationship. Then from there, the professional moves to tackle patient and family needs. Sometimes, when there was a particular problem, the order of the interaction was reversed.

#### We want to know about your experience with the disease

Along with interest in the person, health professionals also showed concerns regarding the trajectory of the disease and the resultant symptoms. The professional’s behaviour was active in solving clinical problems and dealing with patient concerns.

*The team goes for a homecare visit. The daughters of the patient say that their mother has not been sleeping at night. The doctor sits down close to the patient's bed. They both smile. “How are you? (doctor) Someone told me you have not been sleeping well? What's wrong? Are you in pain? Do you still see those animals during the night?” The patient says no. The doctor gives examples that sometimes our head and our heart are too distressed, and this makes sleep difficult (I stand up, touch the patient, and wait in silence). 30 minutes later, the patient says that her daughter, who took care of her, was going to travel to America. Her concern was that she might not see her again.* (Oak, homecare service)

The exploration of the reasons for the problems or symptoms sometimes identifies disease-related aspects and others that are more personal, clarifying professionals’ attempts to address a person as a whole in conducting their work. They even tackle patients’ or families’ wishes during the disease process and talk through decisions to be made, the writing of wills and other personal matters.

*The team’s intervention is oriented to find out how the patient wants to live and die. Today with the family the team discusses the will of a 50-year-old patient who had a stroke. The family says, “Aggressive interventions, no!” The PC team agrees to this plan.* (Oak, inpatient service)

It is frequently observed that the team adapts to the patient, and passes on a message of availability, because they want to care for the patient throughout the trajectory of the disease. It is implicit in their behaviour. PC professionals’ attitudes aim to establish a therapeutic relationship, personal and professional, based on trust and dedicated time.

*Team members (PC) walk quickly around the hospital; a non-stop pace that barely gives them time to eat. They will attend to a patient in the outpatient area. Everyone enters the office and the patient is invited to sit with his family. The doctor asks about the patient’s symptoms and his expectations. It seems like time has stopped. Seated around a table, patient/family and team speak for about 30 min. The team listens to the patient in a focused way, all the questions are answered, fears are acknowledged, and the way they do it is incredibly delicate and open.*

The discussion of the findings of this message with the professionals involved showed that they agreed. They emphasized the purpose of all this: one professional said, “I want to achieve the ultimate purpose, which is clinical. I want to know you as a person not because I am a good person but because we are clinicians” (OAK). This is in alignment with another professional’s comment: “It is essential to know the cause of the suffering of this person to know how best to relieve it. I see her as a person who suffers, and I can help clinically. Knowing her, allow me to identify her internal resources. That may help to deal with the disease.” (Kiri).

Unanimously they relay that all their actions had a transversal intention to know the experience of the patient and his or her preferences and wishes in the achievement of a quality clinical intervention. This was assertively explained, but not always explicitly conveyed in patient interactions. These nuances were crucial for clinicians and implicit in the behaviours and attitudes observed, but rarely shared aloud.

### Message 3 – “family matters: they are also important to us”

The third message that PC professionals convey is that family matters. It is comprised of two sub-themes: “We are here to relieve the suffering of the family” and “We support the caregivers”.

#### We are here to relieve the suffering of the family

Health professionals proactively try to know more about the patient’s family and their situation. It was observed that they are always alert to signs of suffering.

*The team is going to visit a patient diagnosed with severe dementia. She seems relaxed and almost asleep. Quickly, the physician looks at the caregiver and, without hesitation, goes to meet him. The two turn to the window because the caregiver is crying. The doctor comforts him by placing his arm around his shoulders. They are talking about the patient's condition: "how exhausting is to see these health swings; how difficult it is for her not to be able to do more." The physician continues to listen.* (Almond, inpatient service)

#### We want to support the caregivers

Professionals were commonly observed helping families to take care of the patient, addressing the topic of how to deal with difficult conversations and facilitating the understanding and acceptance of the disease trajectory.

The support message was transversal across all sites; however, it was relayed in different ways. The message was conveyed by an explicitly way (e.g. the team help the family using direct discourse during a conference) or using an implicit way (e.g. the team invite family to other office), as described below:

*In a family conference, the caregiver says she will not tell her mother, who is in residence, that her father is dying. The team sits with her to help her in this difficult decision so (as they later discuss) she does not feel bad about the decision when the father dies.*

*"She has the right to know, don’t you think?" (PC professional) The daughter cries and says, "I do not know what to do." The team wants to help her decide and demonstrates how she could communicate this news: "If your mother does not ask for your father, do not worry. But if she has conscious moments when she asks about him and asks why he does not visit her, we can try to tell her the truth. For example, tell her that he is not well, that he probably will not come so quickly, and so on progressively. If we speak naturally, the dialogue happens."* (Oak, inpatient service)

*There is repeated behaviour towards family members who, being with the patient, are invited to go into a separate room so that the team can understand their concerns. A patient is coming to the consultation with his daughter. The main concern of the patient is to know if he can go on vacation because the other clinicians had told him that he would die in 6 months, and it had been a year. “I do not know what to do.” (patient) While the doctor physically evaluates the patient, the nurse invites the family to go to another office. The nurse realizes that the daughter of the patient does not agree with “vacation” time. The caregiver has the opportunity to transmit her fear: “I thought my father was going to die in six months, but no. If let him to go on vacation, and if anything happens to him, I need to go there. I have no car or money. I am always running to his house; I am tired.”* (Almond, outpatient consult)

Professional help was observed in multiple tasks (e.g. preparing patients medication, recognizing and dealing with feelings). Professionals showed a disposition to help with whatever the family needed. This help ranged from how to dispense medications to the provision of tips and support to cope with challenging situations. One of the everyday situations was helping to families to cope with the end of life of their loved one. The ultimate intention was to intervene and reduce the suffering of all and to make both physical and emotional pain tolerable.

*“He [patient] was already prepared [to die]. (physician talking with the family) The most important thing is that you can be with him because he can hear you even if he does not respond. Imagine a baby brain. Often it interacts, but it does mostly through emotion. His mind does not know how to process information but understands emotions.” The daughter is invited to bring her children (grandchildren) to the hospital. The doctor explains that the children learn from the feelings and that there is no reason for these manifestations of sadness to be hidden. It is essential that the child recognizes that suffering exists and it’s part of life.* (Almond, inpatient service)

The discussion of the findings of this message with professionals showed that they agreed.

We asked PC participants to give us their input on the following messages: “We are here to relieve the suffering of the family”; “We want to support the caregivers”. They are aware of the support role they play and identify it with phrases like: “We empower the family so that the patient is better taken care of and to integrate the family into this whole process. That is, the family knows how to take better care, and that is also satisfactory for them. I do not know if it is a true intention or a consequence... but we think about it.” (Oak) On the other hand, there is an altruistic intention to promote the self-care of the caregiver, even after the patient dies.

*"We are worried about the family in the whole process, but it also worries us later. For example, we send letters of condolences to family members. It's a very clear message."* (Kiri, inpatient service)

## Discussion

This study as far as we know is the only one that studies the messages that PC professionals relay to patients and caregivers during their clinical encounters. It identifies three main messages that have little in common with the messages that are transmitted in the media according to other studies [6, 18]. “We are a team focused on your wellbeing” is a clear message transmitted by health professionals explaining “what they do” rather than “who they are”. The continuous participant observation of the PC teams allowed us to recognize a group of people who solve problems, collaborate, share and adapt to complex situations with a common objective, similar to other studies [19].

According to the literature, the message of a team sometimes reaches the patients and families [20, 21] but not others, particularly when they do not refer to PC professionals using the word “team”, but rather “they (a group of people), he, she” (personal approach), or even the word “PC” referred to by Klarare et al. [22] Interestingly the study findings show that health professionals, in order to help the patient and avoid suffering, use the word PC selectively. It predominates the discourse of clinical daily practice about symptom control with a very selective use of the wording PC. This is in line with a quite generalized trend to name PC services as Pain and Symptom control teams [23]. The concept “Palliative Care” is still predominantly perceived as “death” or “end of life” and linked to the topic of euthanasia [24, 25]. Using the term “palliative care” during clinical conversations can cause distress for patients and families and also healthcare professionals [23]. Professionals’ awareness of this conception of PC made them abdicate their identity to reach the patient and family in order to create a relationship that facilitates patient wellbeing.

Like other tough conversations, this may be about a cultural feature of being afraid of causing increased suffering. However, the evidence shows that explicit conversations do not necessarily take away hope, make people anxious or increase depression. In fact, it is the opposite [26, 27]. In a modern society, where the society is increasingly called to be more involved in decision making, PC professionals have the responsibility to pass along a clear and truthful message [10]. It is known that lack of consensus on what PC is, is a barrier to the implementation of PC worldwide [28]. By avoiding addressing this openly, Palliative Care health professionals may be perpetuating the myths, misunderstanding and lack of awareness of PC [29, 30].

The message of “You matter: we see you as a person”, is in line with previous studies that recognize the importance of knowing the person in an authentic way, which allows the team to assist the patient and family better and perform clinical tasks [31, 32]. Observed professionals did so through active listening, acceptance and dedication towards the patient, adopting strategies to approach the patient and their family to create and maintain a personalized therapeutic relationship [19, 33, 34]. All this recalls Sinclair’s (2018) model on healthcare compassion based on PC professionals’ experience [35]. The message “you matter” is the result of professional’s virtues brought into the clinical encounter, recalling the teachings of Dame Cicely Saunders: “You matter because you’re you, and you matter to the end of your life. We will do all we can not only to help you to die peacefully, but also to live until you die.” [36]

During the feedback session on the findings, some professionals emphasized that they are interested in the person not because they are good people but because of their clinical intention to help and relieve suffering, which was not always explicitly conveyed. Despite this lack of explicitness, studies suggest that patients felt recognized as people in their intimate life story, and this made them realize that PC professionals really cared about them (“They know me/us ... They really care”) [22].

Also, the message that the family is essential was highlighted in our study and conveyed directly to the family caregivers with a proactive attitude. The message was standard in the PC services, although as mentioned above there were differences in how this was done in different settings. This evidence can be found in Sampsom et al. [24] where families pointed out that the PC team provided “emotional work” and they, therefore, felt supported. The messages perceived by the families were categorized into three levels: i) Respect; ii) Refuge; iii) Restorative. In other words, the PC team communicates availability and empathy, providing an environment of understanding, acceptance and recognition [24] in line with what was observed in the current study.

These three messages are conveyed through an attitude of being present, availability and disposition towards the patient and their family, sometimes implicitly. A previous study comparing oncologists and palliative care professionals concludes that the main difference between them is in their patient communication styles. PC professionals are more available to talk about coping with illness, to support caregivers and conduct advance care planning [37]. Other studies show that PC professionals provide security since they were always available and were quick to respond [22]. It would be interesting in future studies to explore the repercussion of how a particular message is conveyed.

Perhaps we should consider the message that PC professionals relay as simply: I am a health professional willing to care for you (person with severe illness and suffering family) and anything that concerns you. I want to help you to live, treating you as a “whole person” and, gradually, helping you adapt to the situation. PC professionals wish to transmit a more explicit message on “who they are” instead of “what they do”, identifying the work there is to be done in reaching a better understanding of the profession.

### Strengths and limitations

This study is unique in tackling the message conveyed by PC professionals in their interactions with patients. Different PC service professionals have been observed throughout their shifts. The data has been analysed by two researchers and findings discussed with participants to enhance credibility. The findings show common messages from three different PC services in different communities in Spain. Caution is needed when considering the transferability of findings despite the appearance of a common culture, as there may be other nuances in the messages of other PC professionals.

## Conclusion

The findings of this study reinforce the complexity of professional messaging. It seems that there is a common culture, based on the same values, on what messages PC professionals transmit to patients and their families. The PC health professionals sacrifice being identified more readily by their profession for the sake of what they perceive as their patients’ wellbeing. PC professionals forgo self-interest to care for the patient in order throughout the trajectory of their illness, to alleviate suffering and to support the patient’s family. These messages are transmitted through availability, disposition and acceptance towards the ill person. However, not naming and not openly discussing the purpose of Palliative Care and its usefulness to patients and their families can perpetuate the myths, misunderstanding and lack of awareness of PC. The conclusions of this study are potentially transversal to other European countries.

## Data Availability

The data that support the findings of this study are available from the University of Navarra, but restrictions apply to the availability of these data, which were used under license for the current study, and so are not publicly available. Data are however available from the authors upon reasonable request and with permission of the Ethics Committee of the University of Navarra.

## Abbreviations

SECPAL: Spanish Association of Palliative Care
PC: Palliative Care
PO: Participant Observation
UNAV: University of Navarra
